# Editorial Notes

**Published:** 1912-06

**Authors:** 


### Editorial Notes.

**Dr. Andrew Carrick and Dr. James Cowles Prichard.**

In our December number Mr. L. M. Griffiths made reference to the meeting of the Bristol Medical Library Society, at which Dr. James Cowles Prichard was elected President. The report of the meeting in *Felix Farley's Bristol Journal* of July 1st, 1837, contained the following:—

"Dr. Symonds rose to move the election of a President. He commenced by saying that he should be doing injustice to his own feelings and to those of the meeting if he passed over in silence the event which had given origin to the resolution which he was about to submit to them; he alluded to the decease of Dr. Carrick. He felt assured that the members would join him in the opinion that from whatever association of his fellow-men Dr. Carrick was removed, and whatever the object of that association—whether benevolent, friendly or professional—he had not gone without leaving a melancholy blank behind him. They could not but lament him when they remembered the kindness of his heart, the suavity of his manners, the dignified simplicity of his deportment, or the strong vein of native good sense, and the intelligence and varied information which characterised his intellect. Dr. Symonds had often heard him say that some of the happiest hours of his life had been spent among his professional brethren, and he (Dr. Symonds) was sure that those who shared those hours must allow that their departed friend had diffused at least as much pleasure as he had received. He (Dr. Symonds) would not attempt to pronounce the eulogy of Dr. Carrick; he must leave it to persons better qualified, and who had enjoyed a longer personal intimacy with Dr. Carrick than he (Dr. Symonds) could boast of. He must leave it to them to recount the many exertions of their late President for the good of others, and to dwell upon the untiring and successful zeal with which he performed his public duties, as well as upon the amiable qualities which marked his conduct in fulfilling all that was incumbent on him in the private relations of society, and often more than was incumbent upon him, for he was not one of those economists in kindness and courtesy who calculate to a nicety just how much or how little may be expected of them in discharging the friendly offices of life. Dr. Symonds begged to be allowed to advert to an occasion when the admirable qualities of Dr. Carrick's mind and disposition were brought under his more immediate observation—the time when this city was visited with cholera. Dr. Symonds made some remarks upon the services of Dr. Carrick both in the Anti-Cholera Association and at the Board of Health; and among other things, mentioned that the only time he had ever seen the remarkable placidity of his friend's mind at all ruffled was when any benevolent scheme which he had at heart for mitigating the evils of that period met with any unreasonable opposition. Dr. Symonds then said that he wished that every member of the profession might run his course as happily and as usefully, and go to his grave so much honoured as a practitioner, so much beloved as a man. This contemplation of their loss, though so slightly alluded to, must leave a cloud upon their thoughts; but, happily, clouds, whether in the physical or in the moral world, had nothing settled in their constitution—they were in their nature transient, and subject to dispersion. He ventured to predict that the cloud then hanging over the Society was about to pass away, and fancied that he could already see it gilded by the light that was about to beam upon their horizon; in other words, he felt assured that, great as their regret must be for the bereavement which had befallen them, the resolution which he was about to move would afford them much consolation, and might in time, perhaps, make them forget their sorrow in their joy. He had the honour—one which he valued most highly—to propose a successor to the vacant chair. He had stated his inability to pronounce the eulogy of the departed; he was no less unfitted to attempt the panegyric of the living—such a panegyric, at least, as might justly be called for by the name which he was about to propose, a name which would shed lustre upon any institution in the whole wide world of science and literature. That name was *Prichard.* He would not attempt to enumerate the several claims of that name on such honours as they had power to offer, nor the several benefits that would accrue to the Society from it. Dr. Symonds then spoke in detail of several of Dr. Prichard's writings, and showed that the authorship of these respectively would entitle him to the direction of associations devoted either to literature or to antiquarian researches, or to philological study, or to the philosophy of life, or to natural history, or lastly, and most importantly, to practical medicine. Dr. Symonds then went on to say: 'But since we are, as I trust we are, a body of men bent upon the cultivation and diffusion of the literature of medical science, a science which either deeply implicates or collaterally touches upon the several departments of knowledge to which I have adverted, where, I again ask, could we find an individual who by his achievements in all these fields has so signally qualified himself to preside over a society devoted to such purposes as our illustrious townsman, Dr. Prichard? For my own part, I do not believe that there exists another person possessed of his singular combination of attainments. If we are desirous of finding his equal in this particular respect, we must go to past times, and even then our search may be unsuccessful, notwithstanding we fall in with a Mead or a Sir Thomas Browne. Those who justly acquire great fame among men must in the same degree reflect honour upon their species. Dr. Prichard has obtained a reputation confined to no country where arts and sciences prevail. How much honour must then he reflect upon the country which numbers him among her sons, how much more upon the province which claims him, and still more upon the city which has been the scene of his labours! Bri tol then may well endeavour to reciprocate in what manner she can the distinction which she receives. Need I remind my fellow-members that in presenting our tribute to Dr. Prichard we are offering it to one whom the most celebrated societies in the world have delighted to honour—royal societies, national academies, time-honoured Universities? I have still fresh in my memory, and it must be vividly remembered by others now present, an august occasion when, under the dome of a magnificent structure (raised upon the munificence of a physician of other days), surrounded by the very flower of the British profession, a Regius Professor of Medicine handed to Dr. Prichard the highest honours which Oxford has it in her power to confer. The building in which we are now met may not be quite so superb as the Radcliffe Library; we may not constitute just such an assembly as I have mentioned; and certainly the proposer of the present resolution is a very different person from his eloquent and accomplished friend, Dr. Kidd; but I am sure that we will yield to none in a hearty and enthusiastic desire to show that we duly appreciate the honour reflected upon us by our fellow-citizen.' Dr. Symonds then moved 'That James Cowles Prichard, M.D., F.R.S., etc., etc., be requested to accept the office of President of this Society.'

"The announcement of Dr. Prichard's name was received with the loudest demonstrations of pleasure.

Mr. Mortimer seconded the proposition, which was carried by enthusiastic acclamation.

**The South-Western Counties' Association for the Permanent Care of the Feeble-minded.**

THE country is gradually awakening to the fact that the problem of dealing with the feeble-minded is one of national importance, and their multiplication, unchecked as at present, a national danger. Pending the law's delays, attempts have been made to grapple with the question by voluntary agencies. The Lancashire and Cheshire Society is one of the best known and most successful. Their farm colony at Sandlebridge is an object lesson as to what can be done by well-directed efforts with moderate means and without legal powers of detention. In November of last year there was founded the South-Western Counties' Association for the Permanent Care of the Feeble-minded, with the Earl of Radnor as President and Miss Norah L. Fry as Honorary Secretary (*pro tem.*). The counties included are Somerset, Dorset, Devon, Cornwall and Wilts. With Somerset it was thought well to include Bristol. A committee for each of these counties is now actively at work. The objects of the Association are to awaken the public to the danger to the community of allowing the multiplication of the feeble-minded to continue unchecked, to obtain statistics as to the numbers of such persons in each county, to press for the necessary legislation, to establish institutions for the care and improvement of the feeble-minded, and to promote their happiness whilst providing for their permanent detention. This last point is absolutely essential; without legal power of detention following upon due certification attempts to check the multiplication of the feeble-minded must be largely futile. So far as the patients themselves are concerned, from their entire lack of initiative, those who have been taken into a farm colony or institution would stay there quite happily all their lives; but it is found that on their reaching adolescence they are too often removed by their relatives for their own selfish purposes. This very necessary power of detention is contained in all three Bills dealing with the feeble-minded now before Parliament, one of which may be expected to become law this Session.

The committee for Somerset (including Bristol), as the result of preliminary but by no means exhaustive investigations, have ascertained that in Somerset there are a large number of uncertified mentally defective persons between the ages of fifteen and fifty years, many of whom are in receipt of Poor Law relief. Returns show that in addition 282 children in attendance at the Elementary Schools in the administrative county are mentally defective, whilst if the schools of Bristol, Bath and the other towns situate in the county are included in the survey, the total number of mentally defective children is at least 809.

It has been shown that the feeble-minded, with whom epileptics should be included, on account of the close connection between the two groups and also for convenience of administration, are at least as prolific as normal persons, and that the rate of increase is for the persons belonging to these two categories to double in number in each generation. Obviously it is futile to attempt to deal effectively with the problem unless this constant influx of abnormal children is prevented. The first measure should therefore be to segregate in farm colonies or similar institutions the feeble-minded between the ages of fifteen and fifty. No doubt this can only be done gradually, and the initial expense would be heavy. The institutions need not, however, be expensively built, for it would not be necessary to make them of a permanent character. The experience at Sandlebridge has shown that such colonies could be partly self-supporting. If such a plan were carried out, even within the area of one county, the production of abnormal children would in ten years be enormously checked, and in thirty would be comparatively to the present time a negligible quantity. By this time also the survivors of those originally cared for would have become old, and could be transferred to homes for the aged. So that, although the initial expense would be considerable, there would soon be a saving to the rates, and by thirty years this saving would be very great. For this reason alone it is reasonable that grants in aid, possibly in the form of loans for a period of years, should be given by the Government. The gain to the nation physically and morally, by checking the constant production of degenerates, is too obvious to need emphasising. Whatever help may be ultimately obtained from Government, it is to voluntary effort that we must look to deal successfully with this urgent question.

The sub-committee for Bristol and Somerset of the South-Western Counties' Association for the Permanent Care of the Feeble-minded are issuing an appeal for help towards the establishment of farm colonies or other suitable institutions for the reception and care of the feeble-minded in this county. They hope to begin the work at once, and if properly supported by those who realise its importance, should be able to carry it to a successful issue.

**Tuberculin and Tuberculosis.**

The specific treatment of tuberculosis by means of tuberculin has lately made an advance in popularity in this country, but hitherto most of the work done on these lines has originated from Germany or America, and the results have largely been published in German.

In the present number of this *Journal* (page 168) will be found a review of a small book on "Tuberculin Treatment," by Doctors Riviere and Morland, which should meet with a warm welcome. The work of men with considerable clinical experience, it is most lucidly and practically written: together with Dr. Camac Wilkinson's book [somewhat marred by its ultra-polemic tone], and the English translations of the works of Professor Sahli and of Doctors Bandelier and Roepke, this book forms a very complete setting forth of the methods adopted by those who have the largest experience of tuberculin treatment.

The division of all cases of tuberculosis into two classes, namely those who are autotoxic, that is to say are already inoculating themselves with their own tuberculin, and those, on the other hand, in whom the disease is localised, is of the first importance; for it must be admitted that hitherto much of the dosage has been work done in the dark, and the enormous difference in dose recommended by various enthusiasts without any very obvious rationale, has given to scoffers an opportunity of which they have not been slow to avail themselves.

While opinion among authorities seems largely to have crystallised of late, there still remain two important points, apart from questions of dosage and reactions, on which opinions strongly diverge. In the first place, admitting that a positive reaction to a subcutaneous injection of tuberculin signifies an infection by tuberculosis, are we to regard such a reaction as an indication for active treatment in every case? It seems we must at present leave this an open question, with a lingering sense of regret that so difficult a problem will not definitely allow of so simple a solution. Again, does the return of power to react to tuberculin in a patient successfully treated with it call for further specific treatment or not ? Here, also, there is much difference of opinion, and this question too must be left open for future settlement.

Now at the time when Dispensaries for Tuberculosis and Sanatoria for Consumptives are likely to be rapidly multiplied the guidance of this new book will be greatly needed.

**Oliver Colley Maurice.**

We regret that this member of the Medical Board of the Winsley Sanatorium has succumbed to an attack of pneumonia in his forty-fourth year. He will be greatly missed at Marlborough where he practised, and the Sanatorium will have lost one of its keenest and most useful friends.

